# DNA Methyltransferase Genes Are Associated with Oral Mucositis and Creatinine Levels in Oncopediatric Patients

**DOI:** 10.3390/genes14061136

**Published:** 2023-05-24

**Authors:** Beatriz Fernandes de Souza, José Maria Chagas Viana Filho, José Nunes de Queiroz Neto, Marina de Castro Coêlho, Ana Maria Gondim Valença, Darlene Camati Persuhn, Naila Francis Paulo de Oliveira

**Affiliations:** 1 Graduate Program in Dentistry, Health Sciences Center, Federal University of Paraíba (UFPB), João Pessoa 58051-900, PB, Brazil; bfs2@academico.ufpb.br (B.F.d.S.); ana.valenca@academico.ufpb.br (A.M.G.V.); 2 Department of Molecular Biology, Center for Exact and Natural Sciences, Federal University of Paraíba (UFPB), João Pessoa 58051-900, PB, Brazil; jnqn@academico.ufpb.br (J.N.d.Q.N.); darlene@dbm.ufpb.br (D.C.P.)

**Keywords:** inflammation, oral mucosa, epigenetic, children, genetic, DNMT, DNA methylation, mucositis

## Abstract

The aim of this study was to investigate the association of single-nucleotide polymorphisms (SNPs) and the DNA methylation profiles of the DNA methyltransferase (*DNMT*) gene family with oral mucositis in children and adolescents with hematologic malignancies treated with methotrexate (MTX^®^). The population was comprised of healthy and oncopediatric patients aged between 4 and 19 years. An evaluation of oral conditions was performed using the Oral Assessment Guide. Demographic, clinical, hematological, and biochemical data were obtained from medical records. Genomic DNA extracted from oral mucosal cells was used for the analysis of polymorphisms in *DNMT1* (rs2228611), *DNMT3A* (rs7590760), and *DNMT3B* (rs6087990) using the polymerase chain reaction-restriction fragment length polymorphism (PCR-RFLP) technique (*n* = 102) and for DNA methylation using the methylation-specific PCR (MSP) technique (*n* = 85). The allele and genotypic frequencies of SNPs did not reveal any differences between patients with or without oral mucositis. An increase in the methylation frequency for *DNMT1* in patients recovered from mucositis was detected. The *DNMT3A* methylated profile associated with the CC genotype (SNP rs7590760) appeared to be connected to higher values of creatinine. In addition, the *DNMT3B* unmethylated profile associated with the CC genotype (SNP rs6087990) appeared to be connected with higher values of creatinine. We conclude that the *DNMT1* methylation profile is associated with the post-mucositis period and that the genetic and epigenetic profiles of *DNMT3A* and *DNMT3B* are associated with creatinine levels.

## 1. Introduction

The family of genes called *DNMT*s encodes DNA methyltransferase enzymes, which add the methyl radical (CH_3_) to DNA. The presence of the methyl radical in DNA configures DNA methylation, an epigenetic mark involved in the control of gene expression. The methyl radical is added to carbon 5 of cytosines that precede guanines (CpG dinucleotides), which generates 5-methylcytosine (5-mC). The functions of DNA methylation involve the repression of retrotransposons, monoallelic expression of imprinted genes, inactivation of the X chromosome in females, and inhibition of gene expression. The presence of the methyl radical reduces or completely inhibits gene expression by preventing the binding of transcription factors or by binding with methyl-binding proteins, which also prevent the binding of transcription factors to DNA [1].

The family of DNMTs in humans is composed of DNMT1, DNMT2, DNMT3A, DNMT3B, and DNMT3L. DNMT2 and 3L do not have a catalytic function and little is known about their biological activities. Although DNMT1 maintains the methylation profile during the cell cycle, DNMT3A and B are known as de novo methylases, as they add CH_3_ without prior methylation marks [2]. In addition to their role in maintaining and establishing new methylation patterns, DNMTs are also related to DNA damage recognition and repair [3].

Epigenetic mechanisms are important in modulating gene expression, which is altered in a variety of diseases [4]. These mechanisms depend on the activity of DNMTs to maintain the DNA profiles and, consequently, gene expression. In this sense, single-nucleotide genetic polymorphisms (SNPs), as well as the methylation profiles of *DNMT* genes, can alter the expression of DNA methyltransferases, which, in turn, can impact the methylation profiles and gene expression. Indeed, studies have shown an association between the SNPs, methylation profiles, and gene expression of *DNMT*s and inflammatory, tumor, and mental conditions [5,6,7,8,9,10].

In the context of chemo-induced oral mucositis (OM), one SNP from this family was studied (*DNMT3B* rs2424913) but no association was detected [11]. Chemo-induced OM is the most frequent inflammatory condition resulting from chemotherapy treatment for hematologic malignancies in children and adolescents [12,13]. This inflammation appears mainly in the first weeks of treatment and is painful and reversible; however, while present, it can impact the patient’s quality of life, especially in cases of severe oral mucositis (SOM). One of the chemotherapeutic agents that is most associated with OM, which is widely used in the treatment of hematological malignancies, is methotrexate (MTX^®^). It works by impacting DNA synthesis and DNA methylation mechanisms by disrupting de novo purine and pyrimidine biosynthesis and preventing the formation of one-carbon donors necessary for DNA methylation [14,15]. Outcomes of chemo-induced OM include a delay in chemotherapy treatment, the use of opioids, and the use of parenteral nutrition, all of which impact the effectiveness of chemotherapy [16].

Genetic polymorphisms in the context of chemo-induced OM in children have already been addressed in genes involved in inflammatory mechanisms, oxidative stress, epigenetics, and MTX^®^ and vitamin D metabolism [11,17,18]. Regarding the methylation profile, only the global and site-specific methylation profiles in the gene involved in vitamin D metabolism have been evaluated [18,19]. Since this disease is multifactorial, many genetic targets still need to be explored. These data, along with clinical, demographic, and nutritional data, can guide personalized approaches and improve a patient’s quality of life [20].

Based on these facts, our objective was to study the potential association of the SNPs in *DNMT1* (rs2228611), *DNMT3A* (rs7590760), and *DNMT3B* (rs6087990), as well as the methylation profiles of these genes, with chemo-induced OM in children and adolescents undergoing treatment for hematologic malignancies with MTX^®^. Our aim was to determine whether genes from the DNA methyltransferase family are related to chemo-induced inflammation. SNPs were chosen based on their potential functional significance and a minor allele frequency (MAF) > 0.30 according to the 1000Genomes database. For the methylation analysis, the promoter region or exon 1 of these genes was chosen because they are known to be related to gene expression.

## 2. Materials and Methods

### 2.1. Ethics and Study Design

This study was approved by the Research Ethics Committee of the Federal University of Paraíba (João Pessoa, PB, Brazil) (Opinion No. 4,878,034) and was carried out in accordance with the Declaration of Helsinki (1964). Patients with hematological malignancies (lymphomas and leukemias) being treated in the oncopediatric sector of Hospital Napoleão Laureano (João Pessoa, PB, Brazil) between July 2018 and April 2022 were invited to participate in this study. Healthy patients being treated at the Private Dental Clinic, (João Pessoa, PB, Brazil) between 2021 and 2022 were also invited to participate in this study. Oral changes were evaluated by a previously calibrated team (kappa = 0.87) using the modified Oral Assessment Guide (OAG) [21].

For the analysis of polymorphisms, 102 cancer patients aged 4 to 19 years with a diagnosis of hematological malignancies who were undergoing chemotherapy treatment involving MTX^®^ and without oral inflammatory conditions prior to treatment were selected. Patients with no record of professional follow-up by the oral care team during treatment, those in isolation or who were intubated or severely debilitated, and those treated with radiotherapy or both radiotherapy and chemotherapy were excluded. For the analysis of the association between the occurrence of OM and SNPs, the patients were divided into two groups: G1 (*n* = 16) included individuals who did not present OM during chemotherapy treatment, and G2 (*n* = 86) was composed of patients who developed OM during chemotherapy treatment. To analyze the association between OM severity and SNPs, the first 60 days of treatment were considered in an attempt to reduce bias due to treatment duration. G3 (*n* = 23) was composed of individuals from G2 with mild or moderate OM and G4 (*n* = 26) was composed of individuals from G2 with SOM. Only G2 patients with a fully completed OAG severity rating scale recorded in their dental records were included in G3 or G4.

For the analysis of DNA methylation, 85 healthy and cancer patients aged between 4 and 19 were selected. The inclusion criterion for healthy patients was the absence of a diagnosis of neoplasms and inflammation in the oral mucosa. The inclusion criteria for cancer patients included a diagnosis of hematological malignancies, undergoing chemotherapy treatment with MTX^®^, and an absence of oral inflammatory conditions prior to treatment. Patients with no record of professional follow-up by the oral care team during treatment, those in isolation or who were intubated or severely debilitated, and those treated with radiotherapy or both radiotherapy and chemotherapy were excluded. To analyze the association between OM and the methylation profiles, the patients were divided into four groups: (1) healthy (*n* = 21): individuals without cancer; (2) no mucositis (*n* = 16): oncopediatric patients without mucositis; (3) under mucositis (*n* = 17): oncopediatric patients with mucositis at the time of sample collection; and (4) post-mucositis (*n* = 31): oncopediatric patients who had recovered from mucositis.

### 2.2. Sample Collection and DNA Extraction

Oral mucosa cells were obtained by rinsing with 6 mL of sterilized 3% dextrose. Sample processing, as well as DNA extraction, was carried out as previously described [22].

### 2.3. Polymorphism Analysis

**Figure 1:**
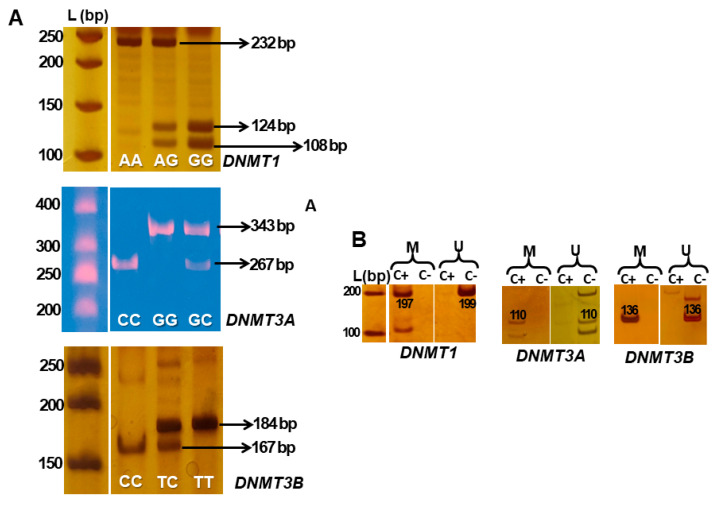
(**A**) Representative fragments of PCR-RFLP reactions for *DNMT*s’ polymorphisms using a 10% polyacrylamide gel stained with either 0.5% silver nitrate (*DNMT1* and *DNMT3B*) or GelRed^®^ (*DNMT3A*). (**B**) Representative bands of control DNAs showing the specificity of the MSP reactions using fully methylated (C+) and fully unmethylated (C−) control DNAs. L (bp) = Ladder (base pair); M = methylated; U = unmethylated.

Analysis of SNPs in rs2228611 *(DNMT1*), rs7590760 *(DNMT3A),* and rs6087990 *(DNMT3B)* was performed using the polymerase chain reaction-restriction fragment length polymorphism (PCR-RFLP) technique, as previously described [23,24] (Table 1). DNA was amplified in 20 μL reactions containing 10 μL of GoTaq^®^ G2 Hot Start Green Master Mix (Promega Corporation, Madison, WI, USA; M7423), forward and reverse primers, 1 µL of DNA, and nuclease-free water. After amplification, enzymatic digestion was performed using restriction enzymes, according to the manufacturer’s recommendations (Thermo Scientific™, Vilnius, Lithuania; ER0031, ER1121, ER1001). The genotypes were evaluated using vertical electrophoresis in a 10% polyacrylamide gel, which was stained with either silver nitrate or GelRed^®^ (Biotium, San Francisco, CA, USA; 41003), and the genotypes were identified according to the band patterns reported in the literature (Figure 1A).

### 2.4. DNA Methylation Analysis

The methylation profile analysis of the *DNMT* genes was performed using the methylation-specific PCR (MSP) technique with primers, as previously described [25,26], except for *DNMT3A*, for which we had our own primer design based on the MethPrimer 2.0 Software (Table 1), according to the DNA sequence found in Genome Browser (chr2:25,227,855–25,342,590). DNA samples (1000 ng) were transformed with sodium bisulfite using the EZ DNA Methylation-Gold kit (Zymo Research, Irvine, CA, USA; D5006), according to the manufacturer’s recommendations. Treated DNA was amplified in 20 μL reactions containing 10 μL of GoTaq^®^ G2 Hot Start Green Master Mix (Promega Corporation, Madison, WI, USA; M7423), forward and reverse primers, 50 ng of bisulfite-transformed DNA, and nuclease-free water. PCR conditions were standardized using control DNAs (EpiTect Control unmethylated DNA, Qiagen, Germantown, MD, USA; 59568 and Universal Methylated Human DNA Standard, Zymo Research, Irvine, CA, USA; D5011) to ensure the specificity of the methylated and unmethylated reactions (Figure 1B). The methylation profiles were evaluated using vertical electrophoresis in a 6% polyacrylamide gel, which was stained with silver nitrate, and categorized into three groups: methylated (amplification of the methylated condition only), unmethylated (amplification of the unmethylated condition only), and partially methylated (amplification of both conditions).

### 2.5. Analysis of Genotypes, DNA Methylation Profiles, Blood Cell Counts, and Biochemical Parameters

Blood cell counts (leukocytes and platelets) and biochemical data (hemoglobin, urea, and creatinine) were obtained from the medical records of oncopediatric patients from the moment of oral mucositis onset. If the patient developed severe oral mucositis during follow-up, data were used from this point onwards. Hematological and biochemical data for patients who did not develop oral mucositis were taken from their last chemotherapy session.

### 2.6. Statistical Analysis

Descriptive and inferential statistical analyses were performed using the free software Jamovi version 1.6 (The Jamovi Project, 2021) with a significance level of 5% and a confidence interval of 95%. *p*-values less than 0.05 were considered significant. Data normality was assessed using the Shapiro–Wilk test and the analysis of the continuous variable of the demographic data was performed using the Kruskal–Wallis test. For each gene, the Hardy–Weinberg (HW) equilibrium was calculated using the Courtlab HW Calculator (COURT, 2005–2008). For the analysis of the association of genotypes, allelic frequency, and methylation status with the occurrence/severity of OM, Fisher’s exact test, Pearson’s Chi-square, or the G test were used. For the analysis of the association between the genotype, DNA methylation profile, blood cell count, and biochemical parameters, the Student’s *t*-test or Mann–Whitney U test was used.

## 3. Results

### 3.1. Demographic and Clinical Data

The population selected for the polymorphism study ranged from 4 to 19 years, with a mean of 10.3 years (±4.78) and the majority being male (53%). No significant differences were found between the groups with or without mucositis (*p* > 0.05; Kruskal–Wallis). Of the 102 patients undergoing chemotherapy, 84.3% developed OM during anticancer treatment. Regarding the underlying diseases, acute lymphoblastic leukemia was the most frequent, affecting 74.5% of patients, followed by acute myeloid leukemia (11.7%), non-Hodgkin’s lymphoma (6.9%), acute promyelocytic leukemia (2.9%), chronic myeloid leukemia (2%), and Hodgkin’s lymphoma (2%). Table 2 shows the demographic and clinical data for each of the groups.

The population selected for the methylation study also ranged from 4 to 19 years, with a mean of 11 years (±4.25) and the majority being female (54.1%). No significant differences were found between the groups of healthy or oncopediatric patients (*p* > 0.05; Kruskal-Wallis). Regarding the underlying diseases for the oncopediatric population, acute lymphoblastic leukemia was the most frequent, affecting 75% of patients, followed by acute myeloid leukemia (9.4%), non-Hodgkin’s lymphoma (6.3%), acute promyelocytic leukemia (4.7%), chronic myeloid leukemia (3.1%), and Hodgkin’s lymphoma (1.6%). Table 3 shows the demographic and clinical data for each of the groups.

### 3.2. Analysis of Polymorphisms

All genotypes were distributed according to the Hardy–Weinberg equilibrium for all groups (*p* > 0.05). For *DNMT1* (rs2228611), the distributions of the alleles and genotypes were similar between the groups, with the A allele and the GA genotype being the most frequent in the population (*p* > 0.05). For *DNMT3A* (rs7590760), the distributions of the alleles and genotypes were similar between the groups, with the G allele and the CG genotype being the most frequent in the population (*p* > 0.05). For *DNMT3B* (rs6087990), the allelic and genotypic frequencies were similar between the groups, with the CT genotype being the most frequent in the population (*p* > 0.05) (Table 4).

### 3.3. DNA Methylation Analysis

**Figure 2:**
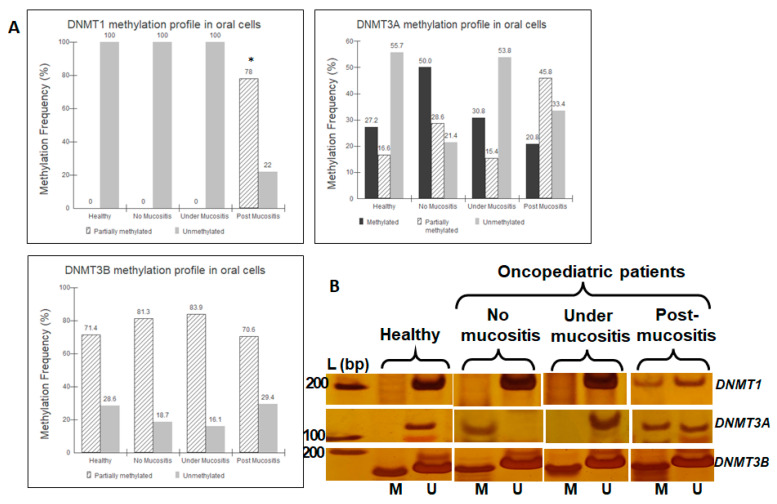
(**A**) Methylation frequency for *DNMT* genes in oral cells of the studied population (*n* = 85). (**B**) Representative bands of groups, showing *DNMT*s’ MSP reactions with a 6% polyacrylamide gel stained with 0.5% silver nitrate. L (bp) = Ladder (base pair); M = methylated; U = unmethylated. *DNMT1* = 197 bp (M) and 199 bp (U); *DNMT3A =* 110 bp (M and U); *DNMT3B* = 136 bp (M and U). * *p* < 0.001; Fisher’s exact.

For *DNMT1*, the post-mucositis group, which consisted of patients whose biological samples were collected after the occurrence of OM, presented a partially methylated profile that was not found in the other groups (*p* < 0.001; Fisher’s exact). For *DNMT3A*, all three profiles were found in the population, without a clear predominance of any particular profile, and no significant differences were found between the groups (*p* > 0.05; G test). Additional analysis comparing the healthy and oncopediatric groups also revealed no significant differences (*p* > 0.05; Fisher’s exact). For *DNMT3B*, the most common profile in the population was found to be partially methylated, with no significant differences found between the groups (*p* > 0.05; Fisher’s exact) (Figure 2).

### 3.4. Analysis of Genotypes, DNA Methylation Profiles, Blood Cell Counts, and Biochemical Parameters

Considering only the oncopediatric population, the combined analysis of the genotypes, DNA methylation profiles, and biochemical parameters showed an association with the creatinine level. The combination of the CC genotype (rs7590760) with the methylated profile for *DNMT3A* was associated with a higher level of creatinine compared to the other combinations of genotypes and methylation profiles (*p* = 0.024; Student’s *t*-test). Likewise, a higher level of creatinine was observed in individuals with the combination of the CC genotype (rs6087990) and the unmethylated profile for *DNMT3B* (*p* = 0.009; Student’s *t*-test) (Table 5).

## 4. Discussion

The pathobiology of OM included mechanisms of oxidative stress, inflammation, and vitamin D metabolism [12,18], and in the present study, our investigation aimed to determine whether the *DNMT* genes involved in the metabolism of the methyl groups are related to this inflammatory condition. DNA methylation is one of the epigenetic markers that orchestrate gene expression so changes in the expression or activity of enzymes that catalyze DNA methylation can impact gene expression, which, in turn, can contribute to disease development [1,2,4].

### 4.1. Polymorphisms

The *DNMT1* gene is located on chromosome 19p13.2, and rs2228611 (G/A) is a synonymous SNP located in exon 17, with a possible regulatory role in alternative splicing. The change from A to G (CCA to CCG) mediates a synonymous mutation at amino acid 463 (proline to proline) and the bioinformatics tool indicated that this SNP is located in the region of the exonic splicing enhancer [27]. This variation leads to the loss of three exonic splicing enhancer binding motifs, which may result in alternative splicing and the generation of multiple transcriptional variants of the *DNMT1* gene [6]. The GG genotype, which was rarely found in our population, has been associated with an increased risk of ovarian cancer and an increased chance of sporadic and recurrent pregnancy loss [24,28], whereas the AA genotype has been associated with schizophrenia and endometriosis [6,29]. However, as in chemo-induced OM, the GA genotype was also the most frequent in a population of women where the relationship of this SNP with the occurrence of miscarriage was studied and no association was detected [30].

The *DNMT3A* gene is located on chromosome 2p23.3, and rs7590760 is located in intron 6, although its influence on gene expression is poorly understood. However, one study showed that patients with acute myeloid leukemia with the CG/GG genotype tended to express *DNMT3A* mRNA 2.38 times higher compared to individuals with the CC genotype. In addition, a survival analysis showed that individuals with lower expression of *DNMT3A* had a lower mean survival (days) compared to those that expressed a higher level of *DNMT3A* [31]. Another study showed that the dominant CC/CG model was associated with susceptibility to noise-induced hearing loss [32]. Interestingly, in the present study, a trend toward a higher frequency of the C allele was observed in the group that developed mucositis (G2), although without statistical significance (*p* = 0.06). This trend could be confirmed by a study with a larger sample. However, the above-mentioned data suggest that the C allele appears to be important and may be associated with disease and lower *DNMT3A* expression. A study of women with endometriosis showed no association with rs7590760, with the majority of the study population having the CG genotype; however, the haplotype study indicated rs7590760 as playing a significant role in the risk of developing endometriosis [33].

The *DNMT3B* gene is located on chromosome 20q11.21, and rs6087990 is located in the −283 promoter region, where the presence of the T allele leads to the loss of the potential Sp1 binding site and is associated with a 50% decrease in promoter activity compared to the C allele [34]. Studies have shown that the CC genotype is associated with a reduced risk of developing colorectal cancer [23] and the TT genotype is associated with an increased risk of developing head and neck cancer [35]. In the present study, the CT genotype was observed to be the most frequent and no significant association was found.

### 4.2. DNA Methylation

Another aspect investigated in the present study was the methylation profiles of *DNMT* genes. Regarding OM, it has already been shown that in oncological children treated with MTX^®^, methylation levels in LINE-1 regions increase during treatment but there has been no relationship with mucositis identified [19]. Another study evaluated the site-specific methylation profile in the *VDR* gene promoter and found no association with OM or chemotherapy treatment [18]. Since the methylation profile is modulated by several factors, including chemotherapy with MTX^®^ [19], a group of healthy subjects who did not undergo chemotherapy was included in this study to determine whether the methylation profiles of *DNMT*s could be used as both an exposure marker and an inflammation marker.

For the *DNMT1* gene, methylated samples in the post-mucositis group were found, unlike the other groups in which only unmethylated samples were found (Figure 2). The same promoter region has been previously studied in the blood cells of adults with acute promyelocytic leukemia, revealing an unmethylated profile in both patients and healthy controls [26]. Two speculations can be made based on the results of the present study: (1) at the time of inflammation, changes are in progress, and only after the resolution can a different established profile be observed; and (2) regeneration of the oral mucosa, post-inflammation, can be regulated by DNA methylation.

A recent study using rats as models of post-ulcer oral mucosa regeneration showed an association between the methylation profile and epithelial regeneration [36]. In contrast to our data, the authors observed a decrease in 5-methylcytosine levels, whereas we found an increase in the methylation level. The only difference between the studies is that Akiyama et al. analyzed the overall methylation profile, whereas we focused on specific sites. Taken together, these data show that oral mucosa regeneration is associated with changes in the DNA methylation profile. Mucosal regeneration includes cell proliferation and differentiation, which are regulated by DNA methylation. This can alter the expression of a variety of genes [2].

Another study looking at lipopolysaccharide-mediated inflammation in human dental pulp cells detected a decrease in *DNMT1* expression in LPS-stimulated cells, hypomethylation in the promoter of the pro-inflammatory cytokine IL6 gene, and an increase in its expression [37]. In the present study, we found *DNMT1* methylation in the group recovered from mucositis, which could be associated with a decrease in its expression since methylation is involved in the regulation of gene expression. Notably, in another study by our group, we found hypomethylation in the promoter of the pro-inflammatory cytokine *TNF-α* gene and also in the group recovered from mucositis [38]. Taken together, these data suggest that DNMT1 plays an important role in inflammation and may be associated with the hypomethylation of pro-inflammatory genes.

For the *DNMT3A* gene, all three profiles were found in the population, without a clear predominance of any particular profile, and no significant differences were found between the groups (Figure 2). There are few studies that address the methylation profile of *DNMT3A*, which makes a comparison difficult. One study evaluated umbilical cord cells collected at birth from babies of women exposed to arsenic and found increased levels of methylation in *DNMT3A* [39]. In the present study, we compared healthy individuals who had not undergone chemotherapy with oncopediatric patients and observed no significant differences between the groups (*p* > 0.05; Fisher’s exact).

One possible explanation for the heterogeneity observed in the profiles of *DNMT3A* is that although our oncopediatric population was homogeneous in terms of hematological neoplasms and treatment with MTX^®^, it was not homogeneous in terms of the type of neoplasm or chemotherapy regimen, which may have included drugs other than MTX^®^. It is possible that the methylation profile varies according to the hematologic malignancy, as observed in myelodysplastic syndrome, where a hypomethylated profile was detected in intragenic regions of *DNMT3A* in bone marrow cells compared to healthy individuals [8]. In addition, it is possible that the chemotherapy regimen is associated with specific changes in the *DNMT3A* gene, as shown in a variety of genes in gastric cancer chemotherapy studies [40].

Thus, although our data do not show differences in relation to the variables studied, they show that the CpG sites of *DNMT3A* studied here are likely modulated by factors that cannot be clarified since there was considerable variation observed among individuals. The data on both polymorphisms and DNA methylation in the *DNMT3A* gene proved to be the most challenging and curious, suggesting that this gene could be further explored in inflammatory and tumor conditions.

For the *DNMT3B* gene, the partially methylated condition was found to be the most frequent, and no significant differences were detected between the groups (Figure 2). The promoter region addressed in the present study has been previously evaluated and an increased frequency of methylation has been observed in patients with hepatocellular carcinoma compared to healthy subjects [25]. In a study of women with endometrial cancer, the authors observed no significant differences between the methylation profiles of individuals with and without endometrial cancer [9]. In the context of inflammatory diseases, most studies address DNMT3B expression and not its methylation profile. The same pattern is found in studies evaluating the effects of chemical agents on this gene. Thus, we conclude that the partially methylated profile observed in the oral cells of children and adolescents is common and has no association with inflammation or exposure to MTX^®^.

### 4.3. Genotype, DNA Methylation Profile, Blood Cell Count, and Biochemical Parameters

When we jointly analyzed the genotype, methylation profile, and hematological and biochemical parameters, we found an association between the genetic, epigenetic, and biochemical profiles.

A simultaneous effect of the rs7590760 polymorphism (CC genotype) and *DNMT3A* methylation on creatinine levels was observed in the pediatric oncology population. Both the genetic and epigenetic profiles of *DNMT3A* converged to decreased *DNMT3A* expression. As already mentioned, one study showed that individuals with the CC genotype had lower *DNMT3A* mRNA expression [31], and hypermethylation has been associated with a decrease in its expression [1]. Thus, this simultaneous effect may have a significant impact on DNMT3A levels and its decreased expression could lead to reduced levels of methylation in genes involved in creatinine metabolism. The association of the genotype and methylation profile with the creatine levels has been previously reported. One study showed an association between hypermethylation and rs1801131 polymorphism in the *MTHFR* gene (methylenetetrahydrofolate reductase) with increased creatinine levels [41].

The same simultaneous effect was observed for *DNMT3B*, where the rs6087990 polymorphism (CC genotype) and the unmethylated profile have been associated with creatinine levels. In this case, both the genetic and epigenetic profiles converge to increased *DNMT3B* expression. The C allele has been associated with the increased activity of its promoter [34] and hypomethylation has been associated with increased expression [1]. Thus, this simultaneous effect may have a significant impact on *DNMT3B* levels and its increased expression could lead to increased levels of methylation in genes involved in creatinine metabolism. One study showed a positive association between the level of global methylation and creatinine, and, in addition, these markers were associated with the *PON*1 (Paraoxonase/Arylesterase Gene) and *IL1β* (Interleukin-1beta) genotype [42].

Interestingly, the simultaneous effects of the genetic and epigenetic profiles of *DNMT3A* and *DNMT3B* are antagonistic in relation to the level of expression: the profile of *DNMT3A* suggests a decrease in its expression and that of *DNMT3B* suggests an increase in its expression, both with similar effects on the level of creatinine, that is, an increase in this biochemical marker of renal function. In fact, little is known about all the functions of each of the DNMTs, which go beyond providing DNA methylation. In addition, sites other than CpGs can be methylated, especially by *DNMT3*, indicating that the role of this family of enzymes is quite complex [1,2]. MTX^®^, on the other hand, is known for its nephrotoxicity, and although an increase in creatinine levels in pediatric patients with hematologic malignancies after treatment with chemotherapy is expected, data from the children in the present study are within the normal range for this condition (0.4 to 1.3 mg/dL) [43]. However, our data suggest that a combination of certain genetic and epigenetic profiles is associated with higher creatinine levels. In [44], the authors found that small variations in creatinine levels, even within the normal range, can impact renal function, leading to a delay in the elimination of MTX^®^, which, in turn, was associated with oral mucositis In the present study, of the eight individuals in whom we found an association between the genetic and epigenetic profiles and the creatinine level, seven had mucositis during chemotherapy treatment.

The limitation of our study is related to its small sample size; however, this was primarily because we studied a population affected by a rare disease (hematological neoplasms), and within this population, we studied chemo-induced oral mucositis, which does not affect all patients. Another issue that had an impact on the sample size was that some patients did not have complete medical records and in these cases, the patient was excluded from some analyses. Despite this limitation, the strength of our work is that it is an unprecedented study focusing on the genetic and epigenetic aspects of a family of genes that remains underexplored in the pathobiology of inflammatory diseases, but that is still of great importance because of its role in the mechanisms that govern gene expression.

Data from studies of genetic and epigenetic markers can be used to create a gene panel, which is a tool for identifying individuals susceptible to the development of certain diseases. In the case of severe oral mucositis, this tool could be useful since it impacts the patient’s treatment and quality of life. The identification of susceptible individuals can guide personalized treatment, to achieve the best results with the least impact on the patient’s quality of life (personalized medicine).

Our data can contribute to the understanding of the molecular mechanisms involved in chemo-induced oral mucositis, as they indicate that the *DNMT* genes are important in this process. In addition, our data raise several questions: (1) Can the *DNMT1* methylation profile observed in children and adolescents who had already recovered from mucositis be reversed? (2) How long does this take? (3) Is this profile associated with *DNMT1* expression? (4) Are the genotype and methylation profile of *DNMT3A* and *3B* associated with the expression profile? (5) Are the genotype and methylation profile associated with the global DNA methylation profile? (6) Is the expression of *DNMT* genes associated with the expression of inflammatory genes? These questions can be addressed through long-term patient follow-up and analyses of the quantification of the mRNA and *DNMT* levels, global methylation profile, and expression of inflammatory genes. In addition, other SNPs and CpG sites in the *DNMT* genes can be investigated.

## 5. Conclusions

We conclude that the *DNMT1* methylation profile is associated with the post-mucositis period. The *DNMT3A* methylated profile associated with the CC genotype (SNP rs7590760) appears to be associated with higher levels of creatinine. In addition, The *DNMT3B* unmethylated profile associated with the CC genotype (SNP rs6087990) appears to be associated with higher levels of creatinine.

## Figures and Tables

**Table 1:** Primers and conditions of PCR reactions for polymorphism (PCR-RFLP) and DNA methylation (MSP) analysis in the *DNMT* genes.

Analysis	Primers (5′-3′)	Annealing (°C)		Product (bp)
**Polymorphism**			**RE-RS** **SNP Location**	
*DNMT1* rs2228611 (G>A)	F: TATGTTGTCCAGGCTCGTCTC R: GTACTGTAAGCACGGTCACCTG	55	*Bsma*I *GTCTC(1/5)*^ exon 17	AA: 232, 28 AG: 232, 108, 124, 28 GG: 108, 124, 28
*DNMT3A* rs7590760 (C>G)	F: TGCTGTGCCTACTCCAAACA R: GCCATGAATGTCCAGAAGGT	62.6	*Rsa*I*GT^AC* Intron 6	CC: 267, 76 CG: 267, 76, 343 GG: 343
*DNMT3B* rs6087990 (T>C)	F: GAAAAAGGCCCCAGAAGGC R: GGCGGGGACGAGGGAAATTT	66	*Ban*I *G^GYRCC* promoter	TT: 184 TC: 184,167, 17 CC: 167,17
**DNA methylation**			**CpG location**	
*DNMT1*	M F: TTAGTAAATCGTGGAGTTTGGAC M R: AACGATAAACGAAAACGACG U F: AGTAAATTGTGGAGTTTGGAT U R: AAAAAACAATAAACAAAAACAACATCT	50 55	promoter	197 199
*DNMT3A*	M F: GGGAGGTATTTGATATCGGTTC M R: AAACTTCGATAACTCCACCTAACG U F: GGGGAGGTATTTGATATTGGTTT U R: AACTTCAATAACTCCACCTAACACT	51 56	exon 1	110 110
*DNMT3B*	M F: GATGATAGGTAGGGGTATCGC M R: CGAACGAAAACACACACACA U F: GTAGGATGATAGGTAGGGGTATTGT U R: ACCCAAACAAAAACACACACAC	59 63	promoter	136 136

RE: restriction enzyme; RS: restriction site; SNP: single-nucleotide polymorphism; M: methylated; U: unmethylated; F: forward; R: reverse.

**Table 2:** Demographic and clinical data of oncopaediatric patients for the study of genetic polymorphisms (*n* = 102).

	G1 No Mucositis during Chemotherapy (*n* = 16)	G2 Mucositis during Chemotherapy (*n* = 86)	G3 Mild/Moderate Mucositis (*n* = 23)	G4 Severe Mucositis (*n* = 26)
Age (years) Mean (±SD)	10.1 (±3.6)	10.4 (±4.9)	8.7 (±4.8)	10.8 (±4.6)
Sex—n (%)				
Girls	11 (68.8)	32 (37.2)	10 (43.5)	12 (46.0)
Boys	05 (31.3)	54 (62.7) *	13 (56.5)	14 (54.0)
Cancer—n (%)				
ALL	11 (68.8)	65 (75.5)	20 (87.0)	19 (73.0)
AML	02 (12.5)	10 (11.6)	02 (9.0)	02 (8.0)
APL	01 (6.3)	02 (2.3)	-	02 (8.0)
CML	01 (6.3)	01 (1.2)	-	-
HL	01 (6.3)	01 (1.2)	01 (4.0)	-
NHL	-	07 (8.2)	-	03 (12.0)

n: absolute frequency; %: percentage frequency; SD: standard deviation; -: missing value; ALL: acute lymphoblastic leukemia; AML: acute myeloid leukemia; APL: acute promyelocytic leukemia; CML: chronic myeloid leukemia; HL: Hodgkin’s lymphoma; NHL: non-Hodgkin’s lymphoma. * *p* < 0.05: Chi-square test.

**Table 3:** Demographic and clinical data of healthy and oncopaediatric patients for the study of DNA methylation (*n* = 85).

	Healthy (*n* = 21)	No Mucositis (*n* = 16)	Under Mucositis (*n* = 17)	Post-Mucositis (*n* = 31)
Age (years) Mean (±SD)	10.2 (3.2)	10.1 (3.6)	10 (4.3)	12.7 (4.7)
Sex—n (%)				
Girls	14 (66.7)	11 (68.8)	7 (41.2)	14 (45.2)
Boys	7 (33.3)	5 (31.3)	10 (58.8)	17 (54.8)
Cancer—n (%)				
ALL	-	11 (68.8)	12 (70.6)	25 (80.6)
AML	-	02 (12.5)	02 (11.8)	02 (6.5)
APL	-	01 (6.3)	-	02 (6.5)
CML	-	01 (6.3)	-	01 (3.2)
HL	-	01 (6.3)	-	-
NHL	-	-	03 (17.6)	01 (3.2)
Without cancer	21 (100)	-	-	-

n: absolute frequency; %: percentage frequency; SD: standard deviation; -: missing value; ALL: acute lymphoblastic leukemia; AML: acute myeloid leukemia; APL: acute promyelocytic leukemia; CML: chronic myeloid leukemia; HL: Hodgkin’s lymphoma; NHL: non-Hodgkin’s lymphoma.

**Table 4:** Genotypic and allelic frequencies: Hardy–Weinberg Equilibrium (HWE) of polymorphisms in *DNMT* genes in oncopediatric patients with or without oral mucositis (*n* = 102). All patients underwent chemotherapy involving MTX^®^.

SNP	No Mucositis during Chemotherapy ^1^ (*n* = 16)	Mucositis during Chemotherapy ^2^ (*n* = 86)	*p*-Valor (1 and 2)	Mild/Moderate Mucositis ^3^ (*n* = 23)	Severe Mucositis ^4^ (*n* = 26)	*p*-Valor (3 and 4)
***DNMT1* (A > G)**			0.541 ^€^			0.458 ^€^
**n (%)**						
AA	06 (37.5)	26 (30.2)		05 (21.7)	10 (38.5)	
AG	09 (56.3)	44 (51.2)		13 (56.6)	11 (42.3)	
GG	01 (6.3)	16 (18.6)		05 (21.7)	05 (19.2)	
A	21 (65.6)	96 (55.9)	0.302	23 (50.0)	31 (59.6)	0.339 ^¥^
G	11 (34.4)	76 (44.1)		23 (50.0)	21 (40.4)	
HWE (*p*)	0.323	0.729	-	0.531	0.536	-
***DNMT3A* (G > C)**			0.251 ^€^			0.691 ^€^
**n (%)**						
GG	07 (43.8)	23 (26.7)		07 (30.4)	08 (30.8)	
GC	08 (50.0)	45 (52.3)		13 (56.5)	12 (46.2)	
CC	01 (06.3)	18 (20.9)		03 (13.0)	06 (23.1)	
G	22 (71.0)	91 (52.9)	0.062 ^¥^	27 (58.6)	28 (53.8)	0.629 ^¥^
C	9 (29.0)	81 (47.1)		19 (41.3)	24 (46.2)	
HWE (*p*)	0.512	0.642	-	0.426	0.715	-
***DNMT3B* (T > C)**			0.610 ^€^			0.676 ^€^
**n (%)**						
TT	03 (18.9)	26 (30.2)		06 (26.1)	10 (38.5)	
TC	08 (50.0)	39 (45.3)		10 (43.5)	09 (34.6)	
CC	05 (31.3)	21 (24.4)		07 (30.4)	07 (26.9)	
T	14 (43.7)	91 (52.9)	0.341 ^¥^	22 (47.8)	29 (55.8)	0.432 ^¥^
C	18 (56.3)	81 (47.1)		24 (52.2)	23 (44.2)	
HWE (*p*)	0.949	0.404	-	0.536	0.128	-

1–4: G1–G4; n: absolute frequency; %: percentage frequency; -: missing value; €: Fisher’s exact; ¥: Pearson’s Chi-square; HWE (*p*): Hardy–Weinberg equilibrium *p*-value. Note: G3 and G4 were composed only of individuals from G2 with a fully completed OAG severity rating scale recorded in their dental records.

**Table 5:** Analysis of the relationship between the *DNMT* genotypes, DNA methylation profiles, blood cell counts, and biochemical variables in oncopediatric patients.

***DNMT1*** **Genotype**	**Methylated**			**Partially Methylated (*n* = 24)**			**Unmethylated (*n* = 40)**		
		**---**		**AA** **(*n* = 8)**	**AG + GG** **(*n* = 16)**	***p***	**AA** **(*n* = 12)**	**AG + GG** **(*n* = 28)**	***p***
Hemoglobin				9.063 ± 2.536	9.200 ± 1.666	0.874	9.342 ± 1.498	9.721 ± 2.527	0.632
Leukocytes				3850 (1100–6400)	2450 (100–17,500)	0.444	3400 (100–7700)	3900 (900–42,400)	0.244
Platelets				129,000 (54,000–302,000)	134,500 (23,000–533,000)	0.653	140,500 (27,000–415,000)	169,000 (28,000–561,000)	0.384
Urea				17.5 (9.00–33.0)	19.0 (9.00- 38.0)	0.581	16.5 (10.0–61.0)	19.0 (5.10–69.0)	0.545
Creatinine				0.471 ± 0.140	0.512 ± 0.145	0.513	0.454 ± 0.134	0.502 ± 0.172	0.391
***DNMT3A*** **Genotype**	**Methylated (*n* = 16)**			**Partially Methylated (*n* = 17)**			**Unmethylated (*n* = 18)**		
	**GG + GC** **(*n* = 13)**	**CC** **(*n* = 3)**	***p***	**GG + GC** **(*n* = 12)**	**CC** **(*n* = 5)**	***p***	**GG + GC** **(*n* = 14)**	**CC** **(*n* = 4)**	***p***
Hemoglobin	9.238 ± 1.319	10.867 ± 0.473	0.058	10.600 ± 1.923	9.360 ± 1.996	0.249	9.000 ± 2.042	7.550 ± 3.044	0.275
Leukocytes	2900 (1100–42,400)	2200 (1400–3200)	0.239	3950 (100–8000)	6300 (900–7100)	0.712	3250 (100–22300)	1900 (1000–5300)	0.426
Platelets	145,000 (23,000–471,000)	175,000 (56,000–242,000)	1.000	161,500 (41,000–380,000)	152,000 (28,000–302,000)	0.460	118,500 (35,000–561,000)	143,500 (103,000–283,000)	0.721
Urea	17.0 (9.00–35.0)	16.0 (12.0–16.0)	0.417	22.0 (13.0–69.0)	11.0 (10.0–50.0)	0.268	15.0 (5.10–38.0)	19.5 (11.0–31.0)	0.557
Creatinine	0.472 ± 0.112	0.670 ± 0.171	0.024 *	0.482 ± 0.156	0.480 ± 0.175	0.977	0.451 ± 0.136	0.565 ± 0.139	0.162
***DNMT3B*** **Genotype**	**Methylated**			**Partially Methylated (*n* = 50)**			**Unmethylated (*n* = 13)**		
		**---**		**TT + TC** **(*n* = 39)**	**CC** **(*n* = 11)**	***p***	**TT + TC** **(*n* = 8)**	**CC** **(*n* = 5)**	**p**
Hemoglobin				9.230 ± 2.092	10.036 ± 1.871	0.253	9.550 ± 2.7449	9.600 ± 2.440	0.974
Leukocytes				3000 (100–42,400)	3200 (1300–6600)	0.882	2000 (300–7700)	5000 (100–17,500)	0.509
Platelets				154,000 (23,000–561,000)	175,000 (36,000–415,000)	0.267	98,500 (27,000–296,000)	110,000 (35,000–533,000)	0.524
Urea				19.0 (5.10–69.0)	16.0 (10.0–35.0)	0.766	19.0 (9.00–45.0)	16.0 (11.0–61.0)	0.713
Creatinine				0.504 ± 0.170	0.482 ± 0.107	0.688	0.395 ± 0.0961	0.576 ± 0.107	0.009 *

---: methylated profile was not detected for *DNMT1* and *DNMT3B* genes. Hemoglobin (g/dL); leukocytes and platelets (mm^3^); and urea and creatinine (mg/dL). Hemoglobin and creatinine: described by mean, standard deviation, and *p*-value (Student’s *t*-test); leukocytes, platelets, and urea: described by median, minimum-maximum (respectively), and *p*-value (Mann–Whitney U). * Student’s *t*-test.

## Data Availability

All the data are contained in the manuscript and will be made available from the corresponding author upon reasonable request.
